# Opposite Effects of Moderate and Extreme Cx43 Deficiency in Conditional Cx43-Deficient Mice on Angiotensin II-Induced Cardiac Fibrosis

**DOI:** 10.3390/cells8101299

**Published:** 2019-10-22

**Authors:** Laura Valls-Lacalle, Corall Negre-Pujol, Cristina Rodríguez, Saray Varona, Antoni Valera-Cañellas, Marta Consegal, Jose Martínez-González, Antonio Rodríguez-Sinovas

**Affiliations:** 1 Cardiovascular Diseases Research Group, Department of Cardiology, Vall d’Hebron University Hospital and Research Institute, Universitat Autònoma de Barcelona, Departament de Medicina, Pg. Vall d’Hebron 119-129, 08035 Barcelona, Spain; laura.valls@vhir.org (L.V.-L.); corall_np@hotmail.com (C.N.-P.); tonivalerac@hotmail.com (A.V.-C.); marta.consegal@vhir.org (M.C.); 2 Centro de Investigación Biomédica en Red sobre Enfermedades Cardiovasculares (CIBERCV), 28029 Madrid, Spain; CRodriguezS@santpau.cat (C.R.); SVarona@santpau.cat (S.V.); JMartinezG@santpau.cat (J.M.-G.); 3 Institut de Recerca del Hospital de la Santa Creu i Sant Pau, IIB-Sant Pau, 08041 Barcelona, Spain; 4 Instituto de Investigaciones Biomédicas de Barcelona (IIBB-CSIC), IIB-Sant Pau, 08041 Barcelona, Spain

**Keywords:** angiotensin II, fibrosis, collagen, connexin 43, hypertrophy

## Abstract

Connexin 43 (Cx43) is essential for cardiac electrical coupling, but its effects on myocardial fibrosis is controversial. Here, we analyzed the role of Cx43 in myocardial fibrosis caused by angiotensin II (AngII) using Cx43^fl/fl^ and Cx43^Cre-ER(T)/fl^ inducible knock-out (Cx43 content: 50%) mice treated with vehicle or 4-hydroxytamoxifen (4-OHT) to induce a Cre-ER(T)-mediated global deletion of the Cx43 floxed allele. Myocardial collagen content was enhanced by AngII in all groups (n = 8–10/group, *p* < 0.05). However, animals with partial Cx43 deficiency (vehicle-treated Cx43^Cre-ER(T)/fl^) had a significantly higher AngII-induced collagen accumulation that reverted when treated with 4-OHT, which abolished Cx43 expression. The exaggerated fibrotic response to AngII in partially deficient Cx43^Cre-ER(T)/fl^ mice was associated with enhanced p38 MAPK activation and was not evident in Cx43 heterozygous (Cx43^+/-^) mice. In contrast, normalization of interstitial collagen in 4-OHT-treated Cx43^Cre-ER(T)/fl^ animals correlated with enhanced MMP-9 activity, IL-6 and NOX2 mRNA expression, and macrophage content, and with reduced α-SMA and SM22α in isolated fibroblasts. In conclusion, our data demonstrates an exaggerated, p38 MAPK-dependent, fibrotic response to AngII in partially deficient Cx43^Cre-ER(T)/fl^ mice, and a paradoxical normalization of collagen deposition in animals with an almost complete Cx43 ablation, an effect associated with increased MMP-9 activity and inflammatory response and reduced fibroblasts differentiation.

## 1. Introduction

Connexins are a family of membrane proteins with a characteristic structure consisting of four transmembrane domains, cytoplasmic amino- and carboxi-terminal domains, and an extracellular loop [1]. Hexameric connexin assemblies, known as connexons or hemichannels, dock with connexons from adjacent cells, to form intercellular channels, that put into contact the cytoplasms of neighboring cells. In the heart, these intercellular channels gather at cardiomyocyte poles [2], forming plaques called gap junctions. Gap junctions constitute low resistance pathways that are essential to allow electrical current flow between connected cells, and thus, coordinated cardiac contraction [3].

A total of 21 different connexin genes have been described in the human genome, whereas 20 have been found in mice [4]. Each of these genes code for a different protein, all sharing a similar structure and high homology [4]. Connexin 43 (Cx43) is the most ubiquitous connexin isoform, being widely distributed in most tissues, including cardiac cells (cardiomyocytes, fibroblasts, and endothelial and smooth muscle cells) [5,6].

The best known function of Cx43 in the heart is to allow electrical coupling between neighboring cardiomyocytes [3]. The importance of gap junction channels in electrical coupling is demonstrated by evidence showing that altered gap junction coupling through Cx43 intercellular channels increases arrhythmogenesis [7]. However, Cx43 has additional gap junction-dependent and independent functions [8]. Thus, Cx43 gap junction channels allow the existence of chemical coupling between the cytoplasms of connected cells, permitting transfer of most ions and second messengers [9]. Chemical coupling through Cx43 has been suggested to be involved in propagation of cell death during myocardial ischemia reperfusion, as demonstrated by the fact that gap junction uncouplers reduce infarct size after transient ischemia in different animal models [10]. In the same direction, isolated hearts from transgenic mice devoid of Cx43 had reduced infarctions after transient global ischemia [11,12].

Gap junction-independent functions of Cx43 in the heart include those ascribed to free sarcolemmal hemichannels and mitochondrial Cx43. Free hemichannels at the sarcolemma have been suggested to play a prominent role in paracrine communication and may affect cell death and survival [8,13]. In addition, some data suggest that mitochondrial Cx43 plays a key role in ischemic preconditioning, as protection is abolished when Cx43 translocation to the mitochondria is reduced [11,12].

Less known are the effects of Cx43 on scar formation and collagen deposition, especially in the heart. Whereas in most tissues reduction of Cx43 expression or functionality is associated with beneficial effects on scar formation [14,15,16], data on the role of Cx43 in cardiac remodeling and collagen deposition are not conclusive. Thus, some authors have reported that reduced Cx43 expression in heterozygous Cx43^+/-^ mice attenuates collagen content in infarcted and non-infarcted regions and reduces ventricular remodeling and scar area (i.e., infarct size) several weeks after permanent coronary ligature [17,18]. In addition, application of the cell-permeable Cx43 carboxiterminal mimetic peptide αCT1, that competitively inhibits the interaction of endogenous Cx43 with zonula occludens-1, to cryoinjured mouse hearts was associated with reduced gap junction remodeling and lower inducibility of ventricular arrhythmias 7 to 9 days after injury [19], and decreased left ventricular volumes 8 weeks later [20]. In contrast to these findings, it has been reported that reduced cellular coupling in Cx43^Cre-ER(T)/fl^ mice, expressing 50% of normal Cx43 content, associates with increased collagen deposition after pressure overload induced by transverse aortic constriction [21]. However, most of these studies have used mice models with partial Cx43 deficiency (about 50%) present from birth. This fact might have resulted in compensatory changes that may account for some of those findings. In addition, data on scar size might be biased by the acute effects of Cx43 deficiency on cell injury [10]. In this context, the aims of this work were to analyze the effects of an inducible, and almost complete, Cx43 deficiency on cardiac fibrosis and left ventricular remodeling after angiotensin II (AngII)-induced pressure overload in Cx43^Cre-ER(T)/fl^ mice, and to analyze the mechanisms involved.

## 2. Materials and Methods

An expanded description of the methods used in this work can be obtained at the Online Supplement.

### 2.1. Mice and Experimental Protocol

Studies were carried out in adult conditional, Cx43-deficient, Cx43^Cre-ER(T)/fl^ animals and in its Cx43^fl/fl^ littermates (4–6 months old, genetic background: C57BL/6J) of both sexes (sex ratio 1:1). Cx43^Cre-ER(T)/fl^ animals were developed by Eckardt et al [22]. In them, the coding region of one of the Cx43 alleles was replaced by Cre-ER(T), a fusion construct of the Cre recombinase and a specifically mutated domain of the human estrogen receptor. Treatment with 4-hydroxytamoxifen (4-OHT) leads to binding of the drug to the ER(T) domain, inducing Cre activity, whereas ER(T) is insensitive to the natural ligand β-estradiol. Cre activation causes a global deletion of Cx43, after recognition of loxP sites flanking the second Cx43 allele, not modifying expression of other connexin isoforms [7,12,22,23]. Osmotic minipumps were subcutaneously implanted, at the interscapular space, and under 2% isofluorane anesthesia (model 1002, Alzet, DURECT Corporation, Cupertino, CA, USA) to continuously deliver saline or AngII (1000 ng/kg/min, Sigma-Aldrich, St Louis, MO, USA) at a constant flow of 0.25 μl/h, for 14 days [24]. One day after pump implantation, Cx43^Cre-ER(T)/fl^ and Cx43^fl/fl^ mice were intraperitoneally injected with vehicle (oil) or 4-OHT to induce Cx43 ablation in the former, as described before [12,22]. This protocol was chosen to ensure that the reduction in Cx43 content coincided with the time of maximal collagen deposition, and to avoid excessive mortality associated with Cx43 deficiency [7,12,22]. In addition, Cx43^+/-^ mice and their wild-type Cx43^+/+^ littermates (genetic background: C57BL/6J) were used to characterize some of the findings. These animals were a kind gift from Dr. S. Poelzing (Virginia, USA). At the end of the experimental procedure, mice were euthanized with a sodium pentothal overdose (1.5 g/kg, intraperitoneal). Hearts were quickly excised and weighted. The basal half was immediately snap-frozen in liquid N_2_, whereas the apical area was fixed overnight with 4% paraformaldehyde and embedded in paraffin. Cardiac hypertrophy was calculated as the ventricular weight/body weight ratio. Animals were distributed in the following experimental groups: Cx43^fl/fl^ + oil, Cx43^fl/fl^ +4-OHT, Cx43^Cre-ER(T)/fl^ + oil, and Cx43^Cre-ER(T)/fl^ + 4-OHT (n = 8–11/group), together with Cx43^+/+^ and Cx43^+/-^ (n = 4–7/group), either treated with saline or AngII.

To check whether changes in collagen deposition observed in Cx43^Cre-ER(T)/fl^ mice injected with oil after AngII treatment were secondary to increased p38 MAPK activity, 8 additional animals from this group were intraperitoneally treated with 10 mg/Kg/day SB203580, a p38 MAPK inhibitor (prepared at 6.67 mmol/L in 33% DMSO), beginning the first day after pump implantation to the end of the experimental protocol.

### 2.2. Systolic Cardiac Function by Transthoracic Echocardiography

Echocardiographic measurements were performed at baseline and at the end of the experimental protocol, with a Vivid q portable ultrasound system, using an ILS 12 MHz transducer (GE Healthcare, WI, USA), as described [25].

### 2.3. Collagen Content and Cardiomyocyte Size

Cardiac fibrosis was analyzed in paraffin-embedded histological sections (4 μm) stained with Picrosirius Red (Sigma-Aldrich, MO, USA) as described [24]. Mean cardiomyocyte cross-sectional area was measured in sections stained with hematoxylin and eosin [25].

### 2.4. Immunohistochemistry

Immunostaining was performed in paraffin-embedded cardiac sections (4 μm) by overnight incubation (4 °C) with antibodies against LAMP-2/Mac-3 (#sc-19991, 1:200, Santa Cruz Biotechnology Inc., Dallas, TX, USA) or MMP9 (#ab38898, 1:500, Abcam, Cambridge, UK) as previously described [24]. Each staining was performed by duplicate.

### 2.5. Real Time-PCR

Total RNA from frozen mouse myocardium was isolated using the TriPure Isolation Reagent (Roche Diagnostics, Indianapolis, IN) following manufacturer’s instructions [24]. Quantification of mRNA levels was performed by real-time PCR using an ABI PRISM 7900HT sequence detection system (Applied Biosystems, Foster City, CA, USA) and specific primers and probes provided by Applied Biosystems (Assay-on-Demand system) or Integrated DNA technologies (Coralville, IA, USA). Relative mRNA levels were determined using the 2^−ΔΔCt^ method.

### 2.6. Total Myocardial Homogenates

Snap-frozen myocardium was homogenized (Diax 600 homogenizer, Heidolph, Germany) in homogenization ice-cold buffer (in mmol/L: Tris-HCl 20, NaCl 140, EDTA 0.8 (pH 7.8), Tween 20 0.1%, sodium fluoride 1, sodium orthovanadate 1, and a protease cocktail inhibitor (1%)). Protein lysates were obtained from the supernatant after centrifugation at 750 g for 10 minutes (4 °C).

### 2.7. Gelatin Zymography

Enzymatic activities of metalloproteinase (MMP)-9 and MMP-2 in murine cardiac homogenates were measured by zymography, as previously described [26]. Gels were scanned in a CanonScan 9000F scanner and densitometrically analyzed (Image Studio Lite v5.0, LI-COR Corporate, Lincoln, NE, USA).

### 2.8. Cardiac Fibroblasts Isolation

Cardiac fibroblasts were isolated from a pool of 2–4 hearts of adult mice by differential centrifugation of cardiac cells after digestion with a mix of collagenase-trypsin as previously described [24].

### 2.9. Western Blot Analysis

Protein extracts from mouse hearts, and from fibroblasts in culture, were electrophoretically separated on 10% polyacrylamide gels and analyzed by Western blot according to standard procedures [12,27].

### 2.10. Statistics

Data are expressed as mean ±SEM. Statistical analysis was performed using SPSS 15.0 (IBM, Armonk, NY, USA). Differences were assessed by one-way or two-way ANOVA and Tukey post hoc tests. Repeated measures ANOVA (MANOVA) was used for echocardiographic data. Differences were considered significant when *p* < 0.05.

## 3. Results

Two Cx43^Cre-ER(T)/fl^ mice injected with 4-OHT and infused with saline, and three AngII-infused, died during the 14 days interval and were excluded from further analysis. This mortality corresponds to that previously associated with Cx43 deficiency in this model [7,12,22].

### 3.1. AngII Treatment Induces a Similar Hypertrophic Response in Hearts from Cx43^fl/fl^ and Cx43^Cre-ER(T)/fl^ Mice Independently of Cx43 Levels

**Figure 1:**
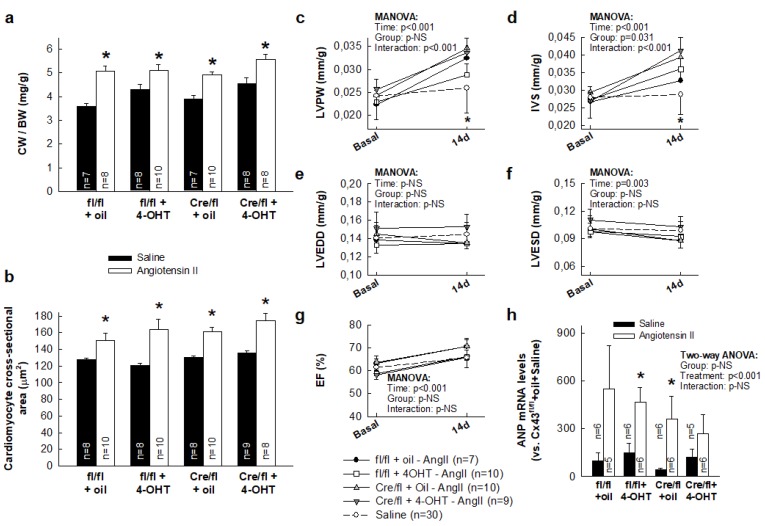
AngII treatment induces a similar cardiac hypertrophic response in hearts from Cx43^fl/fl^ and Cx43^Cre-ER(T)/fl^ mice, independently of Cx43 expression levels. Changes in cardiac weight/body weight (CW/BW) ratio (**a**) and cardiomyocyte cross-sectional area (**b**) in Cx43^fl/fl^ (fl/fl) and Cx43^Cre-ER(T)/fl^ (Cre/fl) mice, treated with saline or angiotensin II for 14 days. * (*p* < 0.05) indicates significant differences vs. the corresponding saline-treated group. (**c**–**g**) Changes in left ventricular posterior wall thickness (LVPW), interventricular septum thickness (IVS), left ventricular end-diastolic internal diameter (LVEDD), left ventricular end-systolic internal diameter (LVESD) (all expressed vs. body weight), and ejection fraction (EF) in the same animals. Data from saline-treated animals are shown pooled as a single group. * (*p* < 0.05) indicates significant differences vs. all groups except Cx43^fl/fl^ +4-OHT + AngII and Cx43^fl/fl^ + oil + AngII in C and D, respectively. (**h**) Changes in myocardial ANP mRNA. * (*p* < 0.05) indicates significant differences vs. the corresponding saline-treated group.

Treatment with AngII for 14 days induced an increase in cardiac weight/body weight (CW/BW) ratio, indicative of cardiac hypertrophy, of similar magnitude in all groups, independently of Cx43 levels (Figure 1a). The lack of influence of Cx43 on the hypertrophic response to AngII was confirmed by assessment of cardiomyocyte cross-sectional area, which was similarly enhanced in all groups (Figure 1b), and by echocardiography, showing increased LVPW and IVS (Figure 1c–d) at day 14, with no changes in ejection fraction, LVEDD, or LVESD (Figure 1e–g). Two-way ANOVA analysis demonstrated a significant effect of AngII infusion (*p* < 0.001) on CW/BW and cardiomyocyte cross-sectional area, with no significant differences between experimental groups and lack of interactions between infusion treatment and group. Consequently, with these data, AngII induced a significant induction of the classic hypertrophic marker ANP in all groups (two-way ANOVA, *p* < 0.001), with no effect of group allocation and lack of interaction between both factors (Figure 1h).

As happened in Cx43^fl/fl^ and Cx43^Cre-ER(T)/fl^ mice, chronic exposure to AngII induced a similar increase in CW/BW ratio in both Cx43^+/-^ animals (3.80 ± 0.13 in saline-treated (n = 4) to 4.8 ± 0.20 mg/g in AngII-infused mice (n = 6), p = 0.004) and in their wild-type littermates (from 4.20 ± 0.09 in saline-treated (n = 7) to 4.60 ± 0.20 mg/g in AngII-treated mice (n = 6), p = 0.04).

### 3.2. AngII Treatment Differentially Modulates Cardiac Fibrosis in Cx43^Cre-ER(T)/fl^ Mice

**Figure 2:**
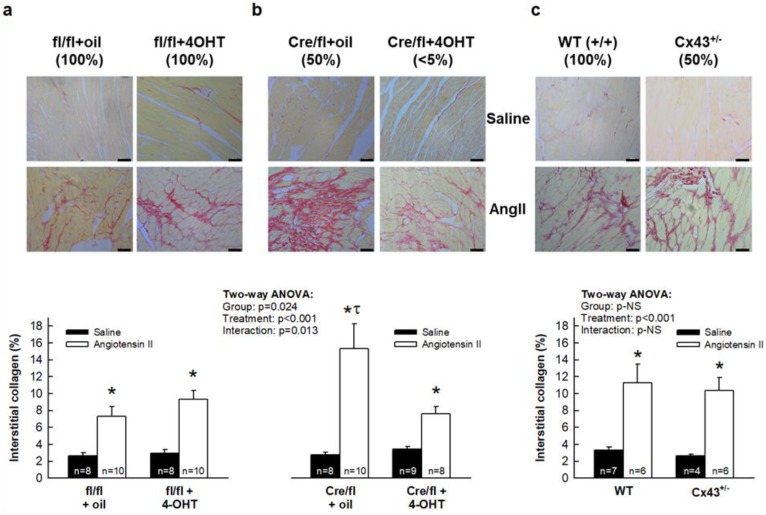
Cardiac fibrosis induced by AngII in Cx43^Cre-ER(T)/fl^ and Cx43^+/-^ mice. Representative images (upper panels) and mean quantification (lower graphs) of interstitial collagen deposition, expressed as percentage of total myocardial area, in Cx43^fl/fl^ ((**a**) fl/fl) and Cx43^Cre-ER(T)/fl^ ((**b**) Cre/fl) mice, after treatment, for 14 days, with saline or AngII. Bar represents 50 μm. (**c**) shows changes in fibrosis in wild-type and Cx43^+/-^ animals. The approximate amount of Cx43 expression is indicated, in parenthesis, below the name of each group. * (*p* < 0.05) indicates significant differences vs. the corresponding saline-treated group. τ (*p* < 0.05) indicates significant differences vs. all Cx43^fl/fl^ or Cx43^Cre-ER(T)/fl^ AngII-treated animals.

Myocardial interstitial collagen was low in all saline-infused experimental groups, with a collagen volume fraction ranging between 2% and 3% (Figure 2). In contrast, treatment with AngII for 14 days increased collagen content in Cx43^fl/fl^ mice injected with oil from 2.67 ± 0.32% to 7.30 ± 1.19% (*p* < 0.05) (Figure 2a). The enhanced collagen deposition in response to AngII was even higher, in oil-treated Cx43^Cre-ER(T)/fl^ animals, expressing 50% of normal Cx43 content (Figure 2b).

To assess whether the marked enhancement in collagen content in response to AngII observed in Cx43^Cre-ER(T)/fl^ + oil mice could be explained by their mild Cx43 deficiency, this approach was also conducted in a mouse model having a similar partial reduction of Cx43 (Cx43^+/-^ mice). Unexpectedly, AngII did not increase collagen deposition in Cx43^+/-^ mice over levels found in AngII-treated Cx43^+/+^ animals (Figure 2c), which may suggest that findings obtained in Cx43^Cre-ER(T)/fl^ + oil mice were, in fact, not directly related to the level of Cx43 expression.

The effects of AngII on collagen deposition observed in Cx43^fl/fl^ mice injected with oil were not modified when animals of this genotype were treated with 4-OHT (Figure 2a). In contrast, the marked enhancement in collagen content observed in mild Cx43-deficient Cx43^Cre-ER(T)/fl^ mice was reverted in animals with an almost complete Cx43 deletion (Cx43^Cre-ER(T)/fl^ treated with 4-OHT) (Figure 2b), with collagen values approaching those found in Cx43^fl/fl^ mice after AngII (Figure 2a). Two-way ANOVA analysis demonstrated significant effects of both group allocation and infused treatment, and a significant interaction between both variables in Cx43^fl/fl^ and Cx43^Cre-ER(T)/fl^ animals, but only for infused treatment in Cx43^+/-^ mice.

Analysis of slices using polarized light microscopy showed a significant increase in mature collagen, after AngII, in all groups except in Cx43^Cre-ER(T)/fl^ animals treated with 4-OHT (Supplemental Figure S1).

### 3.3. Paradoxical Overexpression of mRNAs Coding for Proteins Involved in Collagen Turnover in Cx43-Deficient Mice

**Figure 3:**
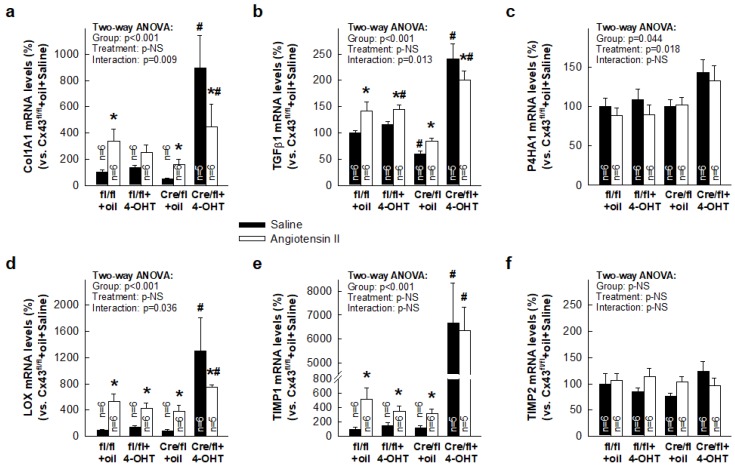
Expression of mRNAs coding for proteins involved in collagen turnover in AngII-treated Cx43^Cre-ER(T)/fl^ mice. Levels of mRNAs coding for proteins involved in collagen synthesis (COL1A1, P4HA1) (**a**–**c**), maturation (LOX) (**d**) and degradation (TIMP1, TIMP2) (**e**–**f**), expressed as percentage vs. Cx43^fl/fl^ + oil mice treated with saline, in both wild-type (Cx43^fl/fl^, fl/fl) and Cx43-deficient mice (Cre/fl), implanted with osmotic pumps containing saline or AngII. * (*p* < 0.05) indicates significant differences vs. the corresponding saline-treated group. # (*p* < 0.05) shows significant differences vs. Cx43^fl/fl^ mice injected with oil and implanted with saline-filled pumps.

Next, we analyzed expression of mRNAs coding for proteins involved in collagen synthesis (COL1A1, TGFβ1, P4HA1), maturation (LOX) and degradation (TIMP1, TIMP2) in both wild-type (Cx43^fl/fl^) and Cx43-deficient mice, infused with saline or AngII. As can be seen in Figure 3, when treated with saline, hearts from Cx43^fl/fl^ mice and from Cx43^Cre-ER(T)/fl^ animals injected with oil (50% Cx43 expression) showed similar levels of all studied mRNAs. In contrast, saline-treated Cx43^Cre-ER(T)/fl^ mice injected with 4-OHT depicted a marked induction of COL1A1, TGFβ1, LOX, and TIMP1. Such induction occurs despite these saline-infused animals did not deposit more interstitial collagen that remaining groups (Figure 2b).

Furthermore, treatment with AngII increased expression of these four mRNAs in Cx43^fl/fl^ mice and in Cx43^Cre-ER(T)/fl^ animals injected with oil, as compared with corresponding mice implanted with saline-containing osmotic pumps, but not in mice with a marked Cx43 deficiency (Cx43^Cre-ER(T)/fl^ +4-OHT) (Figure 3). In this last group, the mRNA levels of COL1A1, TGFβ1, and LOX, although still elevated, were significantly lower in AngII-infused than in saline-infused animals. Two-way ANOVA analysis for COL1A1, TGFβ1, and LOX showed a significant effect of group allocation (*p <* 0.001) and significant interactions with infused treatment (*p <* 0.05), but no effects of AngII treatment. No significant changes were observed for P4HA1 and TIMP2 in any case.

### 3.4. Enhanced Collagen Deposition in Response to AngII in Hearts from Partially Deficient Cx43^Cre-ER(T)/fl^ Mice Correlates with Increased p38 MAPK Activation

**Figure 4:**
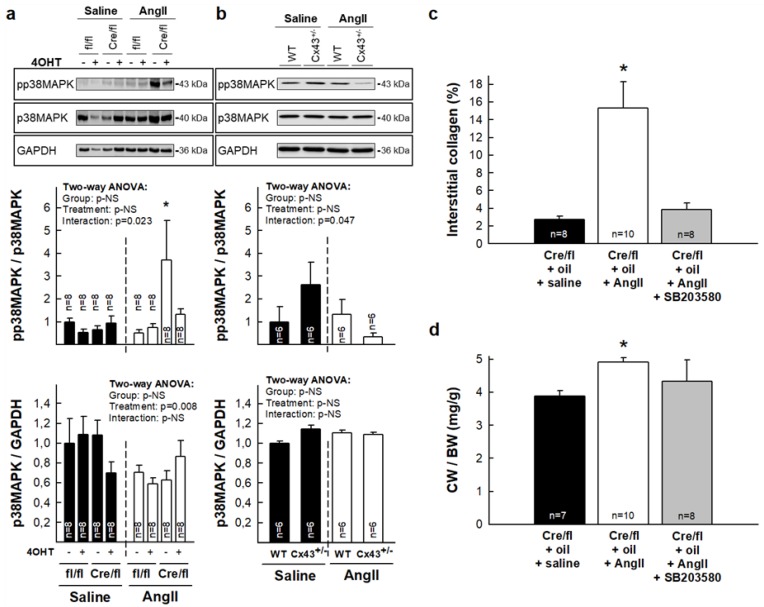
Enhanced collagen deposition in Cx43^Cre-ER(T)/fl^ + oil mice treated with AngII correlates with increased p38 MAPK activation. Expression and degree of activation of p38 MAPK in myocardial samples from Cx43^fl/fl^ (fl/fl) and Cx43^Cre-ER(T)/fl^ (Cre/fl) mice (**a**) and from wild-type (WT) and Cx43^+/-^ animals. (**b**) Upper panels show representative Western blots, whereas middle and bottom panels show degree of activation and total protein levels, respectively. * (*p* < 0.05) indicates significant differences vs. the corresponding control Cx43^fl/fl^ group. (**c**) Interstitial collagen, expressed as percentage of total myocardial area, in hearts from Cx43^Cre-ER(T)/fl^ mice treated with oil and infused, for 14 days, with saline, AngII, or AngII plus the p38 MAPK inhibitor SB203580 (10 mg/Kg/day). (**d**) Changes in cardiac weight/body weight (CW/BW) ratio in the same animals. * (*p* < 0.05) indicates significant differences vs. remaining groups.

AngII signaling is a complex process involving sequential activation of different transduction pathways that regulate extracellular matrix formation, cell survival and proliferation, hypertrophy, and contraction [28,29]. Thus, we tested whether the effects of AngII on collagen deposition in Cx43-deficient mice could be explained by changes in some of these pathways. As depicted in Supplemental Figure S2, there were no changes in the degree of activation (i.e., phosphorylation) of Akt, ERK1/2, STAT3, or SMAD2/3 in any experimental group, neither under saline or AngII infusion, although there was a significant increase, in the last case, in the total amount of ERK1/2 in animals in which Cx43 expression was abolished (Cx43^Cre-ER(T)/fl^ +4-OHT). In contrast, the enhanced AngII-induced collagen deposition seen in Cx43^Cre-ER(T)/fl^ mice receiving oil (50% of Cx43 expression) was associated with an increased p38 MAPK activation (Figure 4a). This enhanced p38 MAPK phosphorylation was not observed in AngII-treated Cx43^+/-^ mice (Figure 4b), which correlated with the unaltered deposition of collagen seen in these animals (Figure 2c).

To check whether the enhanced collagen deposition induced by AngII seen in oil-treated Cx43^Cre-ER(T)/fl^ mice was secondary to increased p38 MAPK activity, a group of these animals were treated with SB203580, a p38 MAPK inhibitor, for the entire experimental period. As seen in Figure 4c, treatment with SB203580 markedly attenuated the enhanced collagen deposition induced by AngII in this group. Similarly, SB203580 reduced the increase in CW/BW ratio induced by AngII in these animals (Figure 4d).

As happened with Akt, STAT3, or SMAD2/3, no changes were observed in the expression of other proteins involved in collagen synthesis (P4HA1) or in myofibroblasts differentiation (α-SMA) in any experimental group, neither under saline or AngII treatment (Supplemental Figure S3a-b). Furthermore, changes in Cx43 expression induced by Cre recombinase insertion and by 4-OHT administration were not modified by AngII treatment (Supplemental Figure S3c). On the other hand, increased vimentin (a fibroblast marker) and LOX levels were observed in mice highly deficient for Cx43 (Cx43^Cre-ER(T)/fl^ +4-OHT) under baseline conditions (Supplemental Figure S3d).

### 3.5. Normalization of Collagen Content in Hearts from Cx43^Cre-ER(T)/fl^ Mice Injected with 4-OHT After AngII Treatment is Associated with Increased MMP-9 Activity

**Figure 5:**
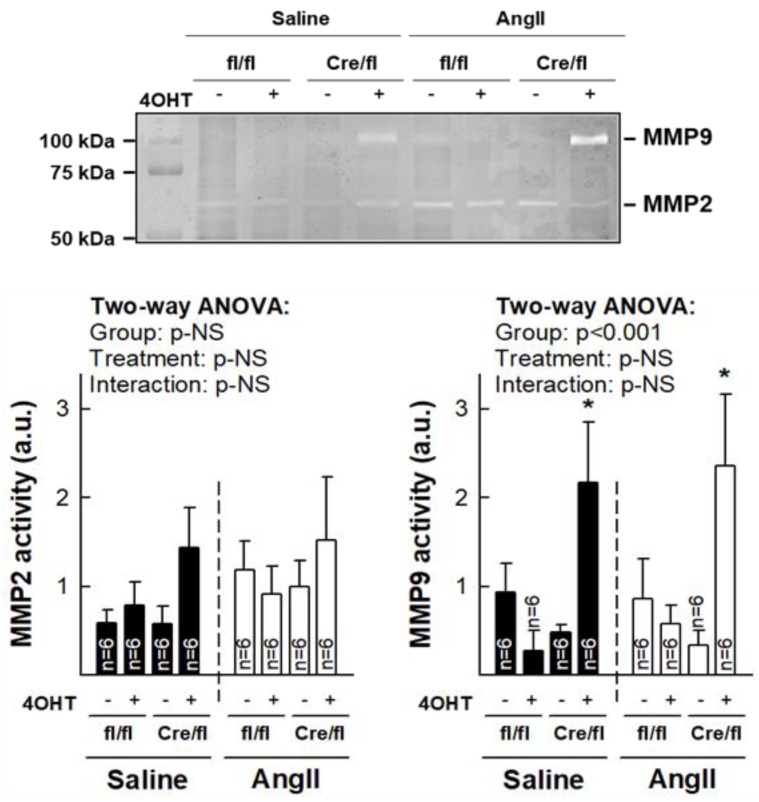
Normalization of collagen deposition in Cx43^Cre-ER(T)/fl^ mice treated with 4-OHT and infused with AngII correlates with increased MMP9 activity. Representative gelatin zymography (upper panel) showing MMP2 and MMP9 enzymatic activity in myocardial samples from saline- and AngII-treated Cx43^fl/fl^ (fl/fl) and Cx43^Cre-ER(T)/fl^ (Cre/fl) mice. Lower panels show mean quantification of 6 different experiments. * (*p* < 0.05) indicates significant differences vs. corresponding the Cx43^fl/fl^ + oil group.

Gelatin zymography of mice myocardial samples demonstrated significantly enhanced MMP-9 activity in animals with a virtual absence of Cx43 (Cx43^Cre-ER(T)/fl^ +4-OHT), as compared with remaining groups, both under saline and AngII treatment (Figure 5). No significant differences were observed for MMP-2 activity.

### 3.6. Isolated Cardiac Fibroblasts from Highly Deficient Cx43^Cre-ER(T)/fl^ Mice Have Altered Phenotype and Differentiation Capacity

**Figure 6:**
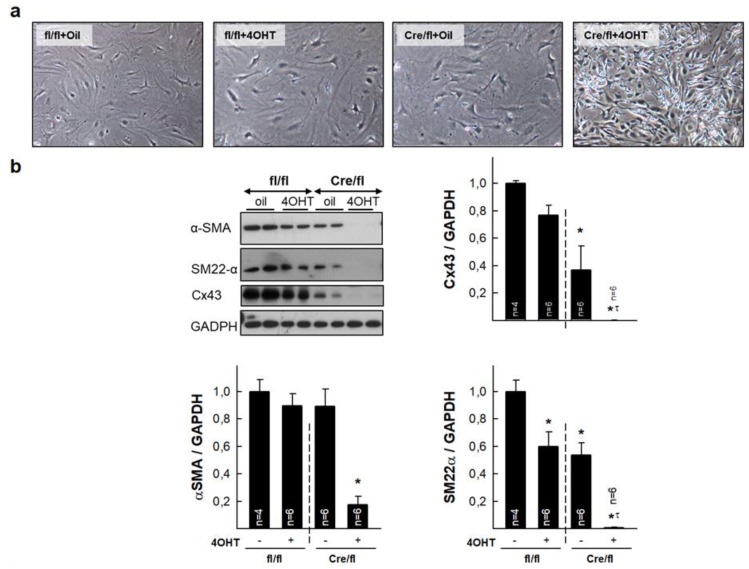
Altered phenotype and expression of differentiation markers in isolated cardiac fibroblasts from Cx43^fl/fl^ and Cx43^Cre-ER(T)/fl^ mice. (**a**) Upper images show morphology of cardiac fibroblasts isolated from control hearts from the four groups of animals. (**b**) Representative Western blots for Cx43, αSMA and SM22α, and total protein expression of analyzed proteins, in fibroblasts isolated from hearts from Cx43^fl/fl^ (fl/fl) and Cx43^Cre-ER(T)/fl^ (Cre/fl) mice. * (*p* < 0.05) indicates significant differences vs. Cx43^fl/fl^ + oil animals. τ (*p* < 0.05) indicates significant differences vs. Cx43^Cre/fl^ + oil animals.

Isolated cardiac fibroblasts from animals with a marked Cx43 deficiency (Cx43^Cre-ER(T)/fl^ +4-OHT) depicted an abnormal phenotype, including reduced size and highly refringent nuclei (Figure 6a). In addition, these cells had reduced expression of α-SMA and SM22α, two markers of cell differentiation (Figure 6b). As expected, Cx43 was absent in cells from these animals, whereas those treated with oil had about half Cx43 expression as compared with cells from Cx43^fl/fl^ mice. Treatment with AngII did not modify expression of these markers in any experimental group (not shown).

### 3.7. Deletion of Cx43 in Cx43^Cre-ER(T)/fl^ Mice Injected with 4-OHT is Associated with Enhanced Cardiac Expression of Inflammatory Markers

**Figure 7:**
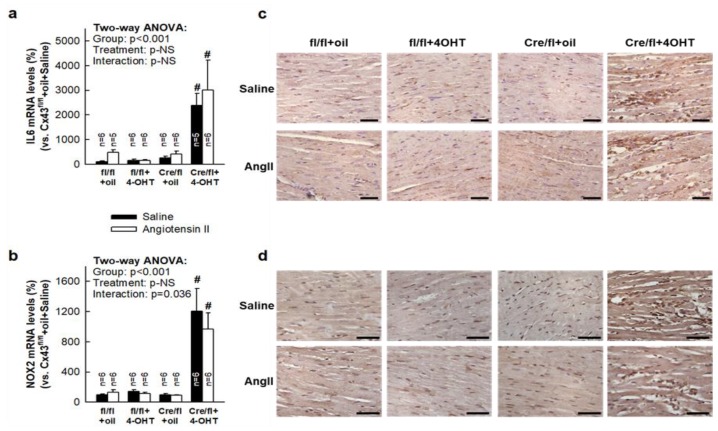
Expression of inflammatory markers in saline- and AngII-treated Cx43^fl/fl^ and Cx43^Cre-ER(T)/fl^ mice. Levels of mRNAs coding for IL-6 (**a**) and NOX2 (**b**), expressed as percentage vs. Cx43^fl/fl^ +oil mice treated with saline, in both Cx43^fl/fl^ (fl/fl) and Cx43-deficient mice (Cre/fl), implanted with osmotic pumps containing saline or AngII. # (*p* < 0.05) shows significant differences vs. Cx43^fl/fl^ mice injected with oil and infused with saline. (**c–d**) Immunohistochemical images of myocardial sections incubated with LAMP-2/Mac-3 (**c**) or MMP9 (**d**) antibodies. Bar represents 50 μm.

Cx43 deletion by OHT treatment of Cx43^Cre-ER(T)/fl^ mice triggered a strong increase in myocardial mRNA levels of IL-6 and NOX2, both in saline- and AngII-infused animals (Figure 7a–b). Two-way ANOVA demonstrated a significant effect of group allocation (p < 0.001), but not of infused treatment and only a marginal significant interaction between both factors for NOX2. Furthermore, immunohistochemical staining of paraffin-embedded cardiac sections demonstrated enhanced expression of LAMP-2/Mac-3 and MMP9 in hearts from Cx43^Cre-ER(T)/fl^ mice treated with 4-OHT under both saline and AngII infusion (Figure 7c–d).

## 4. Discussion

This study demonstrates that ablation of Cx43 expression differentially modulates the fibrotic response to AngII in Cx43^Cre-ER(T)/fl^ mouse hearts. Whereas partial Cx43 deficiency (about 50%) is associated with a huge AngII-induced increase in interstitial collagen deposition in Cx43^Cre-ER(T)/fl^ mice, an almost complete Cx43 ablation (induced by 4-OHT in these mice) reverted this response. The exaggerated collagen accumulation observed in hearts from AngII-infused Cx43^Cre-ER(T)/fl^ mice treated with vehicle, was not evident in Cx43^+/-^ animals, a mice model having a similar mild Cx43 deficiency, which might be indicative that this response is not directly related to Cx43 levels, but was associated with an induction of p38 MAPK signaling, since abnormal AngII-mediated collagen deposition in Cx43^Cre-ER(T)/fl^ mice was abolished by an inhibitor of this pathway. In contrast, normalization of interstitial collagen in 4-OHT-treated Cx43^Cre-ER(T)/fl^ animals, in which Cx43 was almost undetectable, was associated with enhanced MMP-9 activity, reduced fibroblasts differentiation and increased inflammatory reaction.

Chronic exposure to AngII has been shown to cause cardiomyocyte hypertrophy leading to an increase in heart mass [24,28,30,31]. Although cardiac hypertrophy is initially an adaptive process required to sustain cardiac output, it can progress into a maladaptive response triggering cardiomyocyte death and fibrosis, which are responsible for reducing contractility and causing diastolic dysfunction [28,30,32]. In our present study, Cx43^fl/fl^ and Cx43^Cre-ER(T)/fl^ mice treated with AngII for 14 days depicted both cardiac hypertrophy and an increase in collagen deposition. However, whereas cardiac hypertrophy was similarly induced in all the study groups, regardless of Cx43 expression levels, cardiac fibrosis was differentially enhanced. A similar dissociation between cardiac hypertrophy and fibrosis in response to AngII has been previously described for interferon regulatory factor 3 (IRF3) [31]. In our present study, myocardial hypertrophy was determined by the CW/BW ratio, cardiomyocyte cross-sectional area, and by left ventricular dimensions using echocardiography, showing an increase in interventricular septum thickness (IVS) and left ventricular posterior wall thickness (LVPW) in all groups. However, no significant changes were detected in ejection fraction 14 days after AngII treatment, which may indicate that our mice were still in the compensatory phase of left ventricular remodeling and hypertrophy [33].

Most studies, mainly conducted in non-cardiac tissues, have associated a reduction in Cx43 expression or functionality with benefits in scar formation. Research carried out in the skin has demonstrated that down-regulation of Cx43 levels in mice or rats improves the rate of the wound healing effect associated with reduced granulation tissue, and smaller, less distorted, scars [14]. Similarly, hydrogels containing cell-permeable Cx43 carboxiterminal mimetic peptide αCT1, or coated collagen scaffolds with antisense oligodeoxynucleotides targeting Cx43 mRNA, promote regenerative wound healing, reduce the area of scar, and improve skin mechanical properties in mice and pigs [20,34,35,36]. Furthermore, Cx43 deficiency in heterozygous knock-out mice accelerates wound closure and re-epithelization and increases proliferation and activation of dermal fibroblasts [37]. Importantly, in a prospective, multicenter, clinical trial, αCT1 therapy significantly reduced the mean percent ulcer area in patients with chronic venous leg ulcers as compared with conventional compression bandage therapy [38]. However, such benefits are not restricted to the skin. Thus, application of the inhibitory Cx43 mimetic peptide Gap27 has been shown to be effective in promoting healing of superficial epithelial corneal wounds [15]. Furthermore, treatment with TAT-Gap 19, a specific Cx43 hemichannel inhibitor, or carbenoxolone, a general hemichannel and gap junction inhibitor, lowered the degree of liver fibrosis induced with thioacetamide in Balb/c mice [16]. Finally, studies in the heart, involving coronary artery occlusion [17,18] in Cx43^+/-^ mice, or cryoinjury [19,20] in animals treated with αCT1 peptide, have shown attenuation of left ventricular remodeling and reductions in scar area (i.e., infarct size).

Conversely, only few studies have shown a detrimental effect of Cx43 deficiency on collagen deposition and scar formation, and these seem to be restricted to bone and to some concrete scenarios in the heart. Thus, deletion of Cx43 in osteocytes and osteoblasts results in defective bone properties, effects associated with reduced expression of LOX, an extracellular enzyme essential for collagen cross-linking [39]. Regarding the heart, it has been demonstrated that aged Cx43^Cre-ER(T)/fl^ mice, expressing about half of normal Cx43 content, had a higher collagen deposition than its genetic controls Cx43^fl/fl^, an effect that correlated with enhanced arrhythmia inducibility [21]. Furthermore, the increase in collagen content induced by pressure overload through transverse aortic constriction was even higher in young Cx43^Cre-ER(T)/fl^ mice that in Cx43^fl/fl^ animals [21]. Our present study, in which pressure overload was induced using a different strategy, chronic AngII treatment for 14 days, thus, confirms an exaggerated fibrotic response in these animals, having a partial Cx43 deficiency. 

Unexpectedly, when we carried out this experimental approach in Cx43^+/-^ mice, having a similar partial Cx43 reduction, we found that they did not increase collagen deposition in response to pressure overload above levels found in AngII-treated wild-type (Cx43^+/+^) animals. These findings may suggest that data obtained in partially deficient Cx43^Cre-ER(T)/fl^ mice may not be directly related to the degree of Cx43 deficiency. In fact, such exaggerated collagen accumulation observed in hearts from Cx43^Cre-ER(T)/fl^ mice after AngII-induced pressure overload was associated with a marked increase in p38 MAPK activation. Interestingly, the enhanced collagen accumulation was abrogated in the presence of the p38 MAPK inhibitor SB203580. Therefore, our results support that the excessive fibrotic response observed in partially deficient Cx43^Cre-ER(T)/fl^ mice was dependent on the enhanced activation of this pathway. Previous studies have demonstrated that AngII signaling is a complex process involving sequential activation of different protein kinases, including, p38 MAPK [28,29].

In contrast to Cx43^Cre-ER(T)/fl^ mice with partial Cx43 deficiency, Cx43^Cre-ER(T)/fl^ animals with almost a complete ablation of the protein showed a normalized fibrotic response to AngII, which attained values similar to those found in AngII-infused Cx43^fl/fl^ mice. Even more, these mice did not depict the increase in mature collagen observed in remaining groups. This normalization might be understood, when considering this genotype alone, as a protective response against collagen deposition induced by AngII, similar to that described in other tissues when Cx43 expression or function was reduced [14,15,20,34,35,36,37].

To assess the mechanisms involved in the reversion of the exaggerated collagen accumulation seen in 4-OHT-treated Cx43^Cre-ER(T)/fl^ mice, we first analyzed changes in expression of mRNAs coding for proteins involved in collagen synthesis (COL1A1, TGFβ1, P4HA1), maturation (LOX), and degradation (TIMP1, TIMP2). Unexpectedly, we detected a marked increase in mRNAs coding for COL1A1, TGFβ1, LOX, and TIMP1 in these animals, which was already apparent under baseline conditions. This increase appeared despite these saline-infused animals showed very low amounts of interstitial collagen, similar to those found in remaining groups (between 2% and 3%). This response may represent a compensatory mechanism to the increased MMP9 activity and expression seen in these hearts, and correlated with a huge increase in vimentin expression, which is suggestive of a higher amount of fibroblasts in animals with a complete ablation of Cx43. More interestingly, treatment with AngII induced expression of these genes involved in collagen turnover in all groups, but not in Cx43^Cre-ER(T)/fl^ mice treated with 4-OHT. In these animals, mRNAs levels for COL1A1, TGFβ1, and LOX, although still elevated, were significantly lower than those found in its corresponding saline-treated group, and these data correlated with the previously described reduction in collagen accumulation after AngII treatment.

Normalization of the excessive interstitial collagen deposition after AngII infusion in 4-OHT-treated Cx43^Cre-ER(T)/fl^ mice was also associated with a low expression of α-SMA and SM22α and morphological abnormalities in isolated fibroblasts from these animals. Both proteins are markers of the phenotypic switch of fibroblasts to myofibroblasts [24], and these results may be suggestive of reduced differentiation capacity. As cardiac fibroblast to myofibroblasts differentiation has been previously associated with collagen synthesis and secretion [40], these findings may explain, at least in part, the lower collagen deposition in response to AngII observed in these animals. Lack of changes in α-SMA content in tissue extracts, in comparison with isolated fibroblasts, might be due to the fact that, in the heart, this protein is highly expressed in other cell types, mainly smooth muscle cells [41]. 

As mentioned before, Cx43^Cre-ER(T)/fl^ animals injected with 4-OHT had an enhanced MMP-9 activity, assessed by gelatin zymography of mice myocardial extracts, but also an increased inflammatory reaction, denoted by enhanced IL-6 and NOX2 mRNA levels and higher macrophage content. A number of cells within the myocardium express matrix metalloproteinases, including fibroblasts and macrophages [42,43]. A larger number of both cell types might be responsible for increased MMP9 activity and expression, leading to enhanced collagen degradation in these animals.

Previous studies have suggested the presence of either the entire Cx43 protein [44] or its carboxy-terminus [45] in the nucleus, where it may inhibit cell growth [8]. Furthermore, it has been demonstrated that naturally occurring carboxy-terminal fragments of Cx43 can be generated by internal translation of the Cx43 transcript [46], with several isoforms being expressed in the heart [47], including cardiac mitochondria [48]. Functions of these low molecular weight fragments are largely unknown, although some of them can autoregulate full-length Cx43 trafficking [46]. Of note, a recent work has shown that the 20 kDa carboxy-terminal fragment of Cx43 may act as a transcriptional regulator at cell nucleus, binding to N-cadherin promoter and controlling its expression [49]. In view of these findings, one plausible hypothesis to explain our results in 4-OHT-treated Cx43^Cre-ER(T)/fl^ animals infused with AngII is that a lack of these Cx43 fragments would alter transcriptional control in cardiac cells, modifying fibroblast phenotype and increasing the inflammatory reaction, leading in last term to reduced collagen accumulation. This possibility should, in any case, be tested in the future.

Previous works have proposed AT1 receptors to mediate most of the physiologic cardiovascular actions of AngII, including hypertension, vasoconstriction, or the increase in cardiac contractility, among others [29,30]. In addition, stimulation of AT1 receptors would be involved in critical cardiac responses such as cellular hypertrophy, gene reprogramming, and cell death in cardiomyocytes, whereas they mediate cellular proliferation and up-regulation of fibrosis-associated genes in cardiac fibroblasts [30]. Considering the last, it is tempting to speculate that fibrosis in our model should be mediated by AT1 receptors. However, we should also consider that AT2 receptors may have a protective action against fibrosis, as they seem to antagonize, at least under some conditions, AT1-mediated effects [29]. Whether AT1/AT2 receptors could contribute to the fibrotic responses observed in our model is a complex issue that deserves further research. 

In conclusion, this study demonstrates a paradoxical effect of Cx43 deficiency on cardiac collagen deposition induced by AngII in Cx43^Cre-ER(T)/fl^ mice. Whereas a mild Cx43 deficiency is associated with an exaggerated fibrotic response, an almost complete Cx43 ablation after 4-OHT administration normalized collagen accumulation to levels found in wild-type animals (Cx43^fl/fl^). The enhanced collagen deposition seen in hearts from vehicle-treated Cx43^Cre-ER(T)/fl^ mice is dependent on increased p38 MAPK activation, but probably not directly related to changes in Cx43 expression. Further, normalization of the fibrotic response to AngII in animals with very low expression levels of Cx43 is associated with enhanced MMP-9 activity, reduced fibroblasts differentiation capacity, and increased inflammatory response.

## Supplementary Materials

The following are available online at https://www.mdpi.com/2073-4409/8/10/1299/s1, Figure S1: Mature collagen in AngII-treated Cx43^Cre-ER(T)/fl^ and Cx43^+/-^ mice, Figure S2: Expression and degree of activation of Akt, ERK1/2, STAT3 and SMAD2/3 in AngII-treated Cx43^Cre-ER(T)/fl^ mice, Figure S3: Changes in expression of P4HA1, αSMA, Cx43, vimentin, and LOX in hearts from Cx43^fl/fl^ (fl/fl) and Cx43^Cre-ER(T)/fl^ (Cre/fl) mice.
