# Supplementary material for: Opposite Effects of Moderate and Extreme Cx43 Deficiency in Conditional Cx43-Deficient Mice on Angiotensin II-Induced Cardiac Fibrosis
Source: Cells. 2019 Oct 22;8(10):1299. doi: 10.3390/cells8101299 (PMC6830333; doi:10.3390/cells8101299)
Supplement: Supplementary file 1 [file cells-08-01299-s001.pdf]

SUPPLEMENTARY MATERIAL

Opposite Effects of Moderate and Extreme Cx43 Deficiency in Conditional Cx43-Deficient Mice on Angiotensin II-Induced Cardiac Fibrosis

Laura Valls-Lacalle [1,2,†], Corall Negre-Pujol [1,2,†], Cristina Rodríguez [2,3], Saray Varona [2,4], Antoni Valera-Cañellas [1,2], Marta Consegal [1,2], Jose Martínez-González [2,4] and Antonio Rodríguez-Sinovas [1,2,*]

[1] Cardiovascular Diseases Research Group, Department of Cardiology, Vall d'Hebron University Hospital and Research Institute, Universitat Autònoma de Barcelona, Departament de Medicina, Pg. Vall d'Hebron 119-129, 08035 Barcelona, Spain
[2] Centro de Investigación Biomédica en Red sobre Enfermedades Cardiovasculares (CIBERCV), Spain
[3] Institut de Recerca del Hospital de la Santa Creu i Sant Pau, IIB-Sant Pau, 08041 Barcelona, Spain
[4] Instituto de Investigaciones Biomédicas de Barcelona (IIBB-CSIC), IIB-Sant Pau, 08041 Barcelona, Spain
* Correspondence: antonio.rodriguez.sinovas@vhir.org; Tel.: +34 934894038; Fax: +34 934894032
† Both authors contributed equally

Received: 30 September 2019; Accepted: 21 October 2019; Published: date

EXPANDED METHODS

The present study conforms to the NIH Guide for the Care and Use of Laboratory Animals (NIH publications Nº. 85-23, revised 1996), and was performed in accordance with European legislation (Directive 2010/63/UE). The study was approved by the Ethics Committee of our institution (CEEA 49.16).

Mice

Ablation of Cx43 was achieved by 4-hydroxytamoxifen (4-OHT) treatment in adult $Cx43^{Cre\text{-}ER(T)/fl}$ mice (initially these animals had a mixed genetic background 129P2/OlaHsd-C57BL/6J, but they have been extensively crossed with C57BL/6J mices and they can be currently considered as C57BL/6J), as previously described [1,2]. $Cx43^{Cre\text{-}ER(T)/fl}$ animals were developed by Eckardt et al [1]. In them, the coding region of one of the Cx43 alleles was replaced by Cre-ER(T), a fusion construct of the Cre recombinase and a specifically mutated domain of the human estrogen receptor. Treatment with 4-hydroxytamoxifen (4-OHT) leads to binding of the drug to the ER(T) domain, inducing Cre activity, whereas ER(T) is insensitive to the natural ligand β-estradiol. Cre activation causes a global deletion of Cx43, after recognition of loxP sites flanking the second Cx43 allele, with no compensation by overexpression of other connexin isoforms [1-4]. $Cx43^{Cre\text{-}ER(T)/fl}$ mice and their corresponding controls ($Cx43^{fl/fl}$) were injected intraperitoneally with vehicle or 3 mg/day 4-OHT suspended in plant oil for 5 consecutive days. Reduction in Cx43 expression was verified, 13 days after first induction, in all included animals by western blot analysis of cardiac total homogenates [4]. In addition, $Cx43^{+/-}$ mice and their wild-type $Cx43^{+/+}$ littermates (genetic background: C57BL/6J) were used to confirm some of the findings. These animals were a kind gift from Dr. S. Poelzing (Virginia, USA). Genotype of both strains was analyzed by PCR of DNA extracted from tail samples [4].

Experimental protocol

Adult mice (4-6 months old) of all experimental groups and both sexes (sex ratio 1:1) were subcutaneously implanted, at the interscapular space, and under 2% isofluorane anesthesia, with an osmotic minipump (model 1002, Alzet, DURECT Corporation, Cupertino, CA) to continuously deliver saline or angiotensin II (AngII, 1000 ng/kg/min, Sigma-Aldrich, St Louis, MO) at a constant flow of 0.25 µl/h, for 14 days [5]. Anesthetic depth was confirmed by loss of blink reflex and/or lack of response to tail pinch. One day after pump implantation, $Cx43^{Cre\text{-}ER(T)/fl}$ and $Cx43^{fl/fl}$ mice were intraperitoneally injected with oil or 4-OHT to induce Cx43 deficiency, as described before. This protocol was chosen to ensure that the reduction in Cx43 content coincided with the time of maximal collagen deposition, and to avoid excessive mortality associated with Cx43 deficiency [1,2,4]. At the end of the experimental procedure, mice were euthanized with a sodium pentothal overdose (1.5 g/kg, intraperitoneal). Hearts were quickly excised, and after removal of both atria and great vessels, ventricles were weighted and divided in two parts. The basal half was immediately snap-frozen in liquid N_2, whereas the apical area was fixed overnight with 4% paraformaldehyde and embedded in paraffin. Cardiac hypertrophy was calculated as the ventricular weight/body weight ratio. Animals were distributed in the following experimental groups: $Cx43^{fl/fl}$ +oil, $Cx43^{fl/fl}$ +4-OHT, $Cx43^{Cre\text{-}ER(T)/fl}$ +oil, and $Cx43^{Cre\text{-}ER(T)/fl}$ +4-OHT (n=8-11/group), together with $Cx43^{+/+}$ and $Cx43^{+/-}$ (n=4-7/group), either treated with saline or AngII.

To check whether changes in collagen deposition observed in $Cx43^{Cre\text{-}ER(T)/fl}$ mice injected with oil after AngII treatment were secondary to increased p38 MAPK activity, 8 additional animals from this group were intraperitoneally treated with 10 mg/Kg/day SB203580, a p38 MAPK inhibitor (prepared at 6.67 mmol/L in 33% DMSO), beginning the first day after pump implantation to the end of the experimental protocol.

Systolic cardiac function by transthoracic echocardiography

Echocardiographic measurements were performed at baseline and at the end of the experimental protocol, with a Vivid q portable ultrasound system, using a ILS 12 MHz transducer (GE Healthcare, WI, USA) applied to the shaved chest wall of mice lightly anesthetized with isofluorane (1-1.5%). Ejection fraction (EF), left ventricular end-diastolic internal diameter (LVEDD), left ventricular end-systolic internal diameter (LVESD), interventricular septum thickness (IVS) and posterior wall thickness (LVPW) were measured in M-mode recordings. Fractional shortening (FS) was calculated as (LVEDD-LVESD)/LVEDD x 100.

Collagen content and cardiomyocyte size

Cardiac fibrosis was analyzed in paraffin-embedded histological sections (4 μm) stained with Picrosirius Red (Sigma-Aldrich, MO, USA). At least eight areas of the left ventricle per animal were captured using a Leica DM IRBE inverted microscope and a high-resolution digital camera (Leica DFC 550). Images were scanned and evaluated for interstitial collagen content using Image-ProPlus software (Media Cybernetics, MD, USA). The degree of fibrosis was determined as the ratio of interstitial collagen surface area with respect to total myocardial surface area (collagen volume fraction). Quantitative analysis was also performed using polarized light microscopy and refringence analysis to evaluate collagen cross-linking (i.e., mature collagen) as described [5]. Mean cardiomyocyte cross-sectional area was measured in sections stained with hematoxylin and eosin. At least 20 random cells from each heart were measured at x400 magnification [6].

Immunohistochemistry

Immunostaining was performed in paraffin-embedded cardiac sections (4 μm) by overnight incubation (4ºC) with antibodies LAMP-2/Mac-3 (#sc-19991, 1:200, Santa Cruz Biotechnology Inc.) or MMP9 (#ab38898, 1:500, Abcam). Sections were, thereafter, incubated for 1 h (room temperature) with a biotinylated secondary antibody (Vector Laboratories, Peterborough, UK) and then the standard Vectastain avidin-biotin peroxidase complex (ABC; Vector Laboratories, Peterborough, UK) was applied. Colour was developed using 3,3′-diaminobenzidine (DAB) and sections were counterstained with haematoxylin before dehydration, clearing and mounting. Negative controls, in which the primary antibody was omitted, were included to test for non-specific binding. Each staining was performed by duplicate.

Real time-PCR

Total RNA from frozen mouse myocardium was isolated using the TriPure Isolation Reagent (Roche Diagnostics, Indianapolis, IN) following manufacturer's instructions. RNA integrity was determined by electrophoresis in agarose gels and was quantified using a NanoDrop 1000 Spectrophotometer (ThermoFisher Scientific, Waltham, MA). DNase-I-treated total RNA (1 μg) was reverse transcribed into cDNA using the High Capacity cDNA Archive Kit (Applied Biosystems, Foster City, CA) with random hexamers. Quantification of mRNA levels was performed by real-time PCR using an ABI PRISM 7900HT sequence detection system (Applied Biosystems, Foster City, CA) and specific primers and probes provided by Applied Biosystems (Assay-on-Demand system) or Integrated DNA technologies (Coralville, IA) as follows: murine lysyl oxidase (LOX, Mm00495386_m1), prolyl 4-hydroxylase subunit alpha 1 (P4HA1, Mm00803137_m1), tissue inhibitor of metalloproteinases (TIMP) 1 (Mm01341361_m1), TIMP2 (Mm00441825_m1), collagen type I α1 chain (COL1A1; Mm.PT.58.7562513), atrial natriuretic peptide (ANP; Mm.PT.58.12973594.g), interleukin 6 (IL-6) (Mm00446191_m1), cytochrome b-245, β polypeptide (Cybb or NADPH oxidase 2 (NOX2); Mm01287743_m1), and transforming growth factor β (TGFβ1; Mm1178820_m1). As endogenous controls murine TATA-binding protein (TBP; Mm.PT.39a.22214839) and 18S rRNA (4319413E) were used. Similar results were obtained after normalization to both housekeeping genes. Relative mRNA levels were determined using the $2^{-\Delta\Delta Ct}$ method.

Total myocardial homogenates

Snap-frozen myocardium was homogenized (Diax 600 homogenizer, Heidolph, Germany) in homogenization ice-cold buffer (in mmol/L: Tris-HCl 20, NaCl 140, EDTA 0.8 (pH 7.8), Tween 20 0.1%, sodium fluoride 1, sodium orthovanadate 1, and a protease cocktail inhibitor (1%)). Protein lysates were obtained from the supernatant after centrifugation at 750 g for 10 minutes (4ºC).

Gelatin zymography

Enzymatic activities of metalloproteinase (MMP)-9 and MMP-2 in murine cardiac homogenates were measured by zymography. Samples were mixed (1:1) with 2x non-reducing sample buffer (Tris-HCl 0.125 M, SDS 4%, glycerol 20%, bromophenol blue 0.004%, pH 8.8, no boiling). Proteins (30 μg) were resolved in 10% SDS-polyacrylamide gels copolymerized with 1 mg/ml of porcine skin type A gelatin (G1890, Sigma-Aldrich) as a substrate for MMP enzymatic activity, and run at 4°C for 4-5 h. After electrophoresis, the gels were rinsed twice with Triton X-100 2.5% for 15 min at room temperature, and then washed with distilled water. Gels were then incubated in substrate buffer (Trizma base 50 mM, $CaCl_2$ 5 mM, NaN_3 0.02%, ZnCl2 1.02 μM, pH 10) for 20 h at 37°C, and stained with Coomasie Blue 0.5%. Staining was slightly faded in a methanol:acetic acid solution (40%:10%). Areas of gelatinolytic activity appeared as clear bands on a blue background. Gels were scanned in a CanonScan 9000F scanner and densitometrically analyzed (Image Studio Lite v5.0).

Cardiac fibroblasts isolation

Cardiac fibroblasts were isolated from a pool of 2-4 hearts of adult mice by differential centrifugation of cardiac cells after digestion with a mix of collagenase-trypsin as previously described [5]. Cells were maintained in DMEM medium supplemented with 10% FBS, 10 mM L-glutamine, 100 U/mL penicillin/streptomycin, 10 mM L-pyruvate and 2 mM HEPES. Cells were used in the experiments after 2 to 4 passages under control conditions or after treatment for 24 h with 10^{-7} M AngII. Isolation procedure was repeated twice.

Western blot analysis

Protein extracts from mice hearts, and from fibroblasts in culture, were electrophoretically separated on 10% polyacrylamide gels. Expression of Cx43, α-smooth muscle actin (α-SMA), transgrelin (SM22α), vimentin, P4HA1, LOX, TGFβ1, protein kinase B (Akt), extracellular-signal regulated kinase-1/2 (ERK1/2), signal transducer and activator of transcription 3 (STAT3), p38 MAP kinase (p38 MAPK), and Smad2/3 was analyzed by western blot according to standard procedures [7]. Glyceraldehyde-3-phosphate dehydrogenase (GAPDH) was used as loading control. The degree of activation, defined as the ratio between phosphorylated and total forms, was determined for Akt, ERK1/2, STAT3 and Smad2/3. Immunoreactive bands were detected using the SuperSignal West Dura Extended Duration Substrate (Pierce, Rockford, IL, USA) and densitometrically analyzed.

The following antibodies were used: rabbit anti-Cx43 (#C6219, Sigma, dilution 1:8000), mouse or rabbit anti-α-SMA (#A5228 (Sigma, 1:1000) or # ab5694 (Abcam, 1:2000), respectively), rabbit anti-vimentin (#ab92547, Abcam, 1:2000), goat anti-P4HA1 (#ab59497, Abcam, 1:500), rabbit anti-LOX (#NB100-2527, Novus Biologicals, 1:1000), rabbit anti-TGFβ1 (#ab92486, abcam, 1:1000), rabbit anti-Akt (#9272 and #9271, Cell Signaling, 1:1000 and 1:500 for total and phosphorylated (Ser473) forms, respectively), rabbit anti-ERK1/2 (#4695 and #9101, Cell Signaling, 1:1000 and 1:500 for total and phosphorylated (Thr202/Tyr204) forms, respectively), rabbit anti-STAT3 (#9132 and #9134, Cell Signaling, 1:1000 and 1:500 for total and phosphorylated (Ser727) forms, respectively), rabbit anti-p38 MAPK (#8690 and #4511, Cell Signaling, 1:1000 for total and phosphorylated (Thr180/Tyr182) forms, respectively), anti-Smad2/3 (total forms: #sc398844 (raised in mice), Santa Cruz Biotechnology, 1:500; phosphorylated forms (Thr8): #TA325852 (raised in rabbit), Origene, 1:1000), goat anti-transgrelin (SM22α; sc-18513; Santa Cruz Biotechnology, 1:500), and mouse anti-GAPDH (#GTX627408, GeneTex, 1:1000).

Statistics

Data are expressed as mean±SEM. Differences were assessed by one-way or two-way ANOVA and Tukey post-hoc tests. Repeated measures ANOVA (MANOVA) was used for echocardiographic data. Differences were considered significant when $p<0.05$.

SUPPLEMENTAL FIGURE LEGENDS

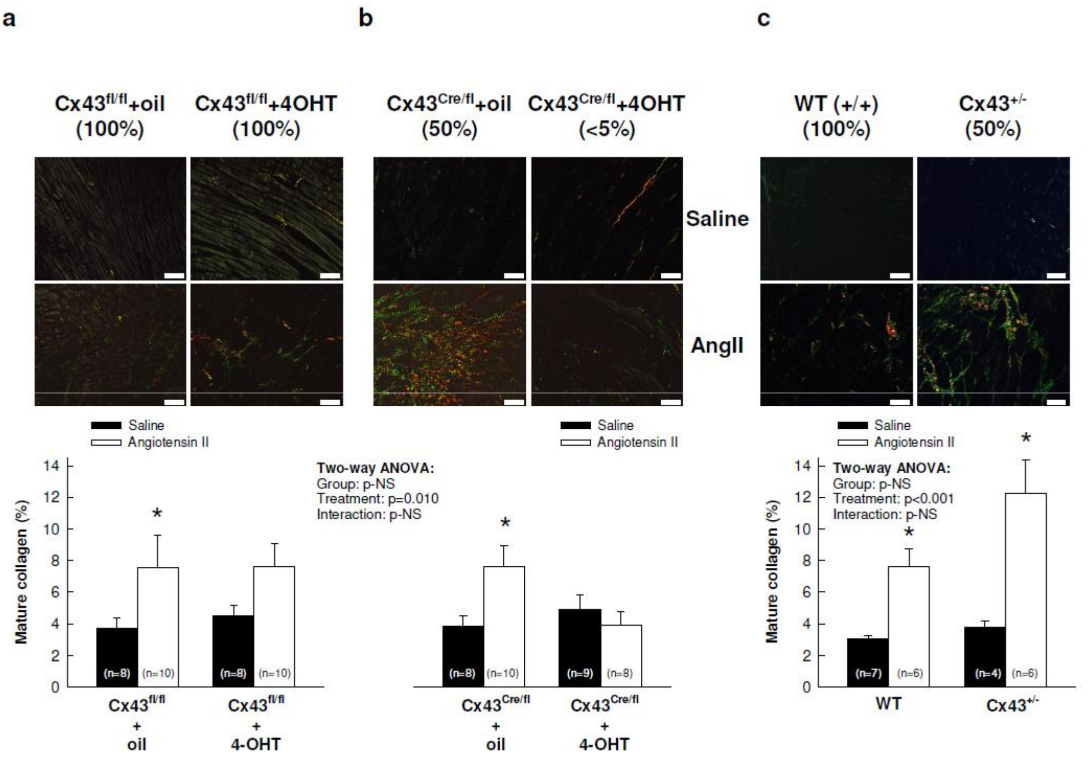

Figure S1. Mature collagen in AngII-treated Cx43$^{Cre-ER(T)/fl}$ and Cx43$^{+/-}$ mice. Representative images (upper panels) and mean quantification under polarized light (lower graphs) of mature interstitial collagen deposition, expressed as percentage of total collagen, in Cx43$^{fl/fl}$ (a, fl/fl) and Cx43$^{Cre-ER(T)/fl}$ (b, Cre/fl) mice, after treatment, for 14 days, with saline or AngII. Mature, highly organized, collagen is stained in red, orange or yellow, whereas low organized collagen appears green. Bar represents 50 µm. (c) shows changes in wild-type and Cx43$^{+/-}$ animals. The approximate amount of Cx43 expression is indicated, in parenthesis, below the name of each group. * ($p<0.05$) indicate significant differences vs. the corresponding saline-treated group.

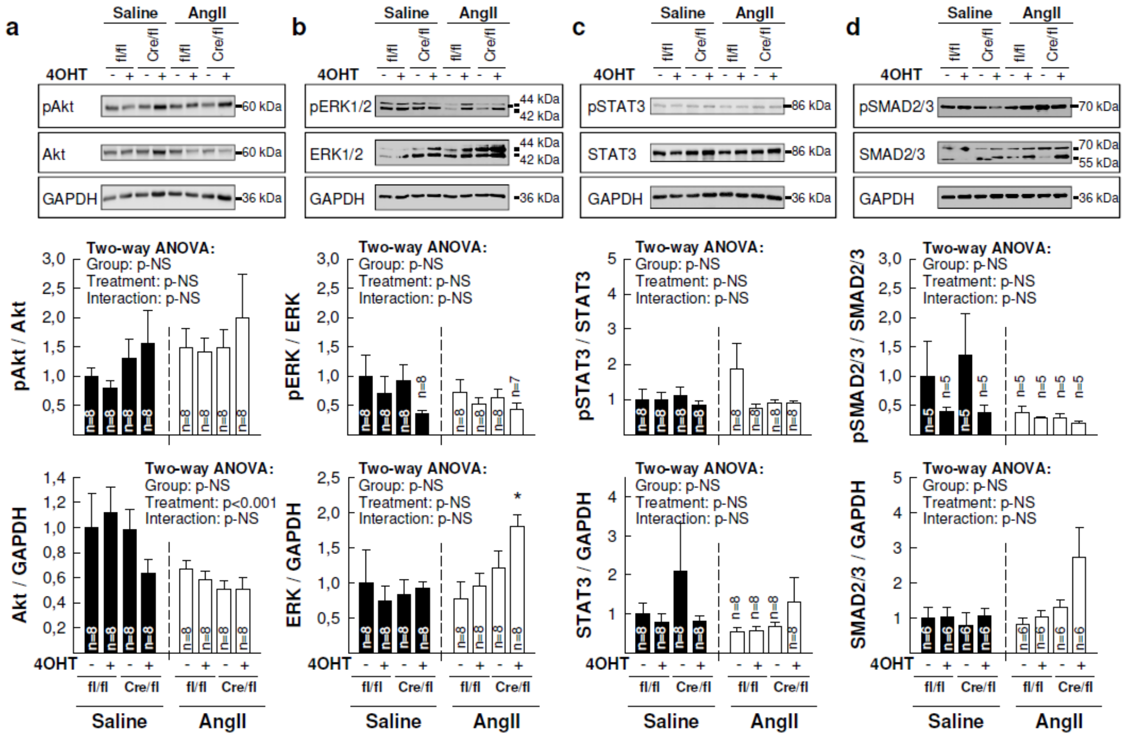

Figure S2. Expression and degree of activation of Akt, ERK1/2, STAT3 and SMAD2/3 in AngII-treated $Cx43^{Cre\text{-}ER(T)/fl}$ mice. Upper panels show representative western blots for Akt (a), ERK1/2 (b), STAT3 (c) and SMAD2/3 (d). Middle and bottom panels show degree of activation (ratio phosphorylated/total forms) and total protein expression of analyzed proteins, in myocardial samples from saline- and AngII-treated $Cx43^{fl/fl}$ (fl/fl) and $Cx43^{Cre\text{-}ER(T)/fl}$ (Cre/fl) mice. * ($p<0.05$) indicate significant differences vs. the corresponding $Cx43^{fl/fl}$ group.

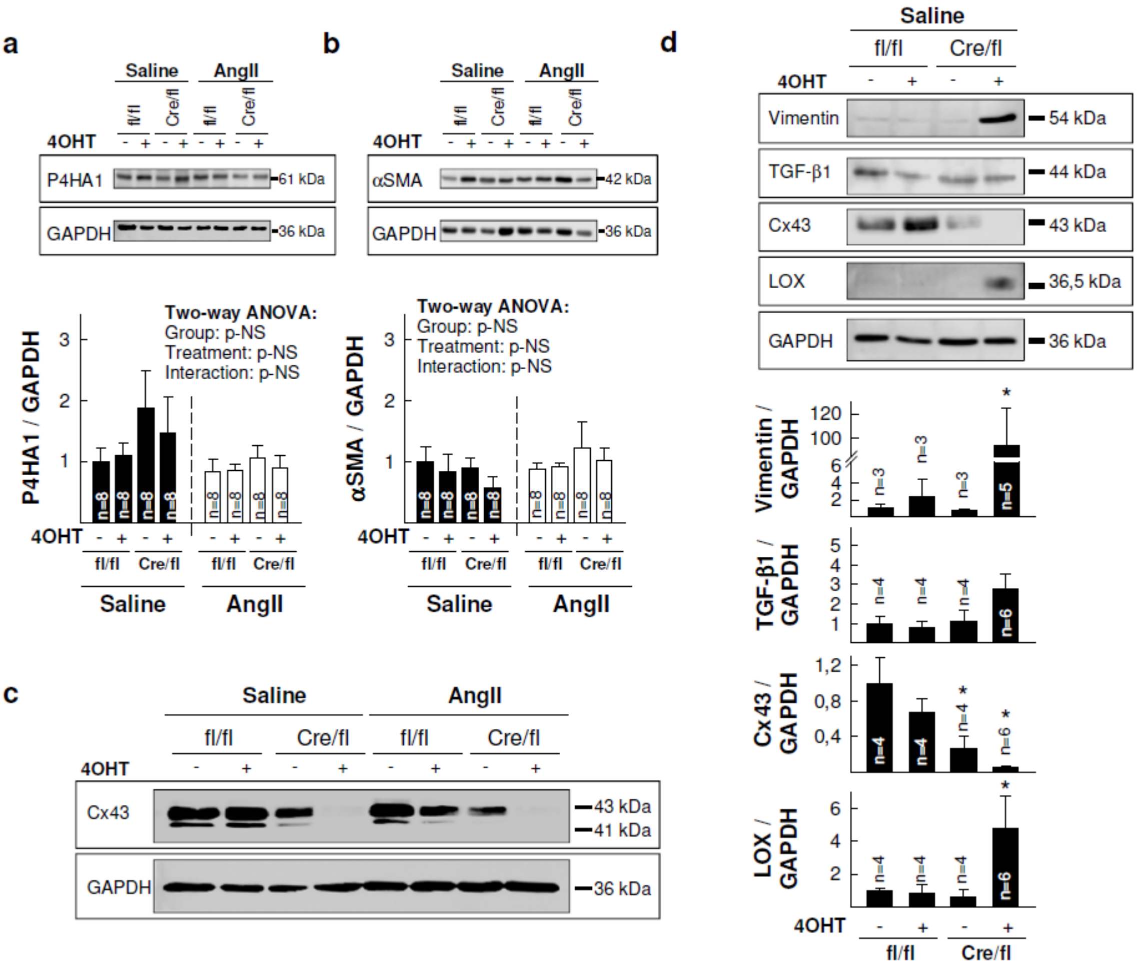

Figure S3. Changes in expression of P4HA1, αSMA, Cx43, vimentin, and LOX in hearts from Cx43$^{fl/fl}$ (fl/fl) and Cx43$^{Cre\text{-}ER(T)/fl}$ (Cre/fl) mice. Upper panels in (a) and (b) show representative western blots for P4HA1 and αSMA in myocardial samples from saline- or AngII-treated mice. Lower panels show quantifications for both proteins. (c) Representative western blot showing changes in Cx43 expression in cardiac samples from saline- and AngII-treated mice. (d) Representative western blots showing changes, under baseline conditions, for vimentin, TGFβ1, Cx43 and LOX. Lower panels show quantifications. * ($p<0.05$) indicate significant differences vs. the Cx43$^{fl/fl}$ group.

References

1. Eckardt, D.; Theis, M.; Degen, J.; Ott, T.; van Rijen, H.V.; Kirchhoff, S.; Kim, J.S.; de Bakker, J.M.; Willecke, K. Functional role of connexin43 gap junction channels in adult mouse heart assessed by inducible gene deletion. *J. Mol. Cell. Cardiol.* **2004**, *36*, 101–110.
2. Sanchez, J.A.; Rodriguez-Sinovas, A.; Barba, I.; Miro-Casas, E.; Fernandez-Sanz, C.; Ruiz-Meana, M.; Alburquerque-Bejar, J.J.; Garcia-Dorado, D. Activation of RISK and SAFE pathways is not involved in the effects of Cx43 deficiency on tolerance to ischemia-reperfusion injury and preconditioning protection. *Basic Res. Cardiol.* **2013**, *108*, 351.
3. van Rijen, H.V.; Eckardt, D.; Degen, J.; Theis, M.; Ott, T.; Willecke, K.; Jongsma, H.J.; Opthof, T.; de Bakker, J.M. Slow conduction and enhanced anisotropy increase the propensity for ventricular tachyarrhythmias in adult mice with induced deletion of connexin43. *Circulation* **2004**, *109*, 1048–1055.
4. Sanchez, J.A.; Rodriguez-Sinovas, A.; Fernandez-Sanz, C.; Ruiz-Meana, M.; Garcia-Dorado, D. Effects of a reduction in the number of gap junction channels or in their conductance on ischemia-reperfusion arrhythmias in isolated mouse hearts. *Am. J. Physiol. Heart Circ. Physiol.* **2011**, 301, H2442–H2453.
5. Galan, M.; Varona, S.; Guadall, A.; Orriols, M.; Navas, M.; Aguilo, S.; de Diego, A.; Navarro, M.A.; Garcia-Dorado, D.; Rodriguez-Sinovas, A.; et al. Lysyl oxidase overexpression accelerates cardiac remodeling and aggravates angiotensin II-induced hypertrophy. *FASEB J.* **2017**, *31*, 3787–3799.
6. Poncelas, M.; Inserte, J.; Aluja, D.; Hernando, V.; Vilardosa, U.; Garcia-Dorado, D. Delayed, oral pharmacological inhibition of calpains attenuates adverse post-infarction remodelling. *Cardiovasc. Res.* **2017**, *113*, 950–961.
7. Boengler, K.; Dodoni, G.; Rodriguez-Sinovas, A.; Cabestrero, A.; Ruiz-Meana, M.; Gres, P.; Konietzka, I.; Lopez-Iglesias, C.; Garcia-Dorado, D.; Di Lisa, F.; et al. Connexin 43 in cardiomyocyte mitochondria and its increase by ischemic preconditioning. *Cardiovasc. Res.* **2005**, *67*, 234–244.
